# Instruments used to assess gender-affirming healthcare access: a scoping review protocol

**DOI:** 10.12688/hrbopenres.13689.1

**Published:** 2023-02-20

**Authors:** Seán Kearns, Philip Hardie, Donal O'Shea, Karl Neff

**Affiliations:** 1 UCD School of Medicine,, University College Dublin,, Belfield,, Dublin 4, D04 V1W8, Ireland; 2 National Gender Service,, St Columcille's Hospital,, Loughlinstown, Co.Dublin, D18 E365, Ireland; 3 UCD School of Nursing, Midwifery and Health Systems, University College Dublin, Belfield, Dublin 4, D04 V1W8, Ireland

**Keywords:** Transgender, Non-binary, healthcare access, healthcare navigation, accessibility, scoping review, study protocol, quantitative

## Abstract

Background: Internationally, the demand for gender-affirming care has increased exponentially in recent years. The clinical presentation of those seeking care has changed with an increase in transmasculine and non-binary identities and a decrease in the average age of those seeking care. Healthcare navigation remains complicated for this population and warrants further investigation in light of ongoing changes in the field. This paper presents a protocol for a methodological scoping review that aims to systematically map and synthesise the extent and nature of the peer-reviewed, published academic literature on the instruments used to assess factors relating to healthcare navigation and healthcare access for transgender and non-binary individuals seeking gender-affirming healthcare.

Methods: This review will search databases (PsychINFO, CINAHL, Medline, and Embase.) and grey literature sources. In line with the methodological framework for scoping reviews, the following six stages will be undertaken: (1) identifying the research question, (2) identifying relevant studies, (3) study selection, (4) charting the data, (5) collating, summarising and reporting results and (6) consultation. The PRISMA Extension for Scoping Reviews (PRISMA-ScR): checklist and explanation will be utilised and reported. The research team will undertake the study as outlined in this protocol and an expert panel of young transgender and non-binary youth will oversee the project through patient and public involvement.

Conclusions: This scoping review has the potential to inform policy, practice, and future research through enhanced understanding of the complex interplay of factors that impact healthcare navigation for transgender and non-binary people seeking gender-affirming care. The results from this study will inform further research into healthcare navigation considerations generally and will inform a research project entitled “Navigating access to gender care in Ireland—a mixed-method study on the experiences of transgender and non-binary youth”.

## Introduction

The World Professional Association of Transgender Health (WPATH) describes the goal of gender-affirming care as partnering with transgender and gender-diverse individuals and working in a holistic manner to address social, mental, and medical health needs ( Coleman *et al.*, 2022). This supportive work is often across the lifespan of an individual from youth to older age and includes fostering a space to explore and question gender openly without influence towards a particular intervention ( Spencer *et al.*, 2021). Due to the complexity of this work, it is best delivered in a multidisciplinary manner, and care should be individually tailored to the individual and their specific needs. Medical interventions may include hormone blockers, cross-sex hormones, and surgical procedures ( Coleman *et al.*, 2022).

As our understanding of gender grows, there is a recognition that gender-diverse presentations may be binary or non-binary and either may seek gender-affirming care inclusive of medical and/or surgical interventions. ( Monro, 2019; Scandurra *et al.*, 2019). In recent years there has been an unprecedented increase in demand for gender-related healthcare and clinical presentations have changed with an increase in natal females and a decrease in the age of presentation to services ( Thompson *et al.*, 2022). Due to the evolving nature of this field of work, continued research is needed to seek to understand the dynamic landscape of gender healthcare.

Currently, gender-related healthcare is typically delivered through either centralised or decentralised models of care ( Koehler *et al.*, 2021). Centralised models of care are described as institutions that provide all or most gender-related services at one location or as one team. In contrast, decentralised care is described as care delivered by multiple different autonomous services. In Europe, the term “gender clinics” or “gender identity clinics “ are commonly used to describe centralised services. There is little evidence of preferred models of care and Koehler *et al.* (2021) detail reasonable pros and cons of both models.

Regardless of the model, the benefits of accessing gender-affirming endocrine care are well documented in adult populations ( Hughto & Reisner, 2016). Some studies have claimed these benefits extend to adolescents receiving puberty suppression ( de Vries *et al.*, 2011) but researchers note the limitations of this evidence due to the lack of high-quality longitudinal studies in this area ( The Cass Review, 2022). Research on outcomes from surgical interventions reports an increase in QoL and improvement in mental health, but again lacks robust longitudinal data ( Swan *et al.*, 2022).

This study is particularly interested in exploring access to gender-related healthcare. The study will seek to explore and understand how healthcare is assessed in this population.

Various theories have been proposed to explain health-seeking behaviours in this population. Minority Stress has been frequently referenced as a source of social stress, due to stigma and discrimination, that can impair healthcare access and mental health ( Hunter *et al.*, 2021). The Gender Affirmation Model has been referenced as a potential protective factor, describing how access can improve when healthcare professionals are known to be affirming and supportive of a person’s gender identity ( Hidalgo *et al.*, 2013). The Anderson healthcare utilisation model has been used to describe barriers related to healthcare access in gender-diverse youth that encompass multiple factors, including social stressors ( Kearns *et al.*, 2021).

Prior qualitative systematic reviews have reviewed elements of healthcare access. McCann *et al.* (2021) examined the psychosocial support needs of transgender and non-binary people highlighting the impact of stigma, discrimination, and marginalisation as well as the importance of formal and informal support systems. Chong *et al.* (2021) conducted a review of the experiences and perspectives of transgender youth in accessing gender care and discussed systemic barriers and feelings of fear and uncertainty in the context of decision-making for youth and parents.

However, to the best of the authors’ knowledge, no quantitative review of the literature regarding healthcare access has been completed to date. Therefore, the authors propose a scoping review to:

i) ascertain a broad overview of the established quantitative literature,
ii) examine how the research has been conducted,
iii) map the results thematically,
iv) identify gaps in the evidence base that can inform future studies.

Due to the nature of this research topic, natal sex and gender will be different from one another. For the purposes of reporting, the authors will disaggregate by gender identity and this will be clearly described, where possible.

## Methods

This scoping review is informed by methods outlined by Arksey and O’Malley (2005) and includes enhancements by Levac *et al.* (2010) and the Joanna Briggs Institute (2015). The framework consists of five iterative stages including 1.) identification of the scoping review question, 2.) identifying relevant studies, 3.) selection of eligible studies, 4.) charting the data, and 5.) collating, summarising and reporting of the results. There is an optional sixth stage which involves consulting with key stakeholders. The research team feels consultation is crucial with populations that are often omitted from research that studies them. The research team will therefore include this sixth stage and utilise an already-established expert panel to consult with key stakeholders. The expert panel consists of ten young adults who have lived experience of being transgender or non-binary and interfacing with health systems. They have reviewed the research question and will be consulted with throughout the stages below. Lastly, they will help to disseminate the results to community members and key stakeholders.

### Stage one: Identification of the scoping review research question

Initially, the literature was read broadly to ascertain the current research priorities and gaps. The Preferred Reporting Items for Systematic reviews and Meta-Analysis extension for Scoping Reviews (PRISMA-ScR) Checklist framework guidelines will guide the systematic scoping review ( Tricco *et al.*, 2018). The research teams’ own background and work in the clinical area aided the questioning. In developing the research question, the mnemonic population, concept, and context framework guided the formulation of the study components ( Peters *et al.*, 2020). In this study, transgender and non-binary individuals seeking gender-affirming care are the identified population, the concept is a healthcare accessibility instrument, and the context is gender-related healthcare access.

The overall aim of the scoping review is to identify, explore and map the existing literature pertaining to healthcare access for transgender and non-binary individuals. The clinical team wishes to compare the literature base between adults and youth. Specifically, the research team is interested in identifying the quantitative instruments used to measure barriers to care.

Thus, the main research question guiding this review is:

*What factors help and hinder access to gender-related healthcare and how are these factors identified by quantitative instruments?*

**Inclusion and Exclusion criteria:** This scoping review will consider quantitative studies that assess healthcare access. The studies must include an instrument (i.e. a questionnaire) and access must be related specifically to gender-affirming healthcare. There is no limit on the years published but the included studies must be in English. For full inclusion and exclusion criteria see Table 1.

**Table 1.** Inclusion and exclusion criteria.

	Inclusion	Exclusion
**Population**	• Trans and non-binary population (All ages)	• LGBT population as opposed to solely transgender population • Healthcare practitioner perspective • Student perspectives
**Concept**	• Accessibility instrument relating to **accessing gender** ** care** • Quantitative study in nature • Mixed-method included if quant data can be isolated	• Not a healthcare accessibility instrument • Study topic not related to accessing gender care • Qualitative in nature
**Context**	• Any geographical location • Setting: Medical centre that provides gender affirming care • Published from: No limit on publication date*	• Setting: If not related to gender affirming care but related to general healthcare
**Types of** ** evidence ** **sources**	• Research studies	• Abstracts • Reviews (systematic, scoping, meta-analysis) • Editorials, commentaries and opinion papers • Dissertations/theses, conference papers
**Language**	• Articles available in English	• Non-English articles

**Study objectives:**

The objectives of this scoping review are to:

1. Identify the instruments/tools used in assessing healthcare for transgender and non-binary individuals seeking gender-affirming care.
2. Compare instruments geographically.
3. Determine the theories underpinning the research.
4. Ascertain the most commonly reported barriers to healthcare navigation.
5. Describe typical methodological aspects, such as study design and outcomes.
6. Identify potential gaps in the research.

The research will examine the above aims in both adult and youth cohorts. Ultimately, the findings will be used to inform the development of a comprehensive healthcare navigation instrument for transgender and non-binary healthcare seekers.

### Stage two: Identifying relevant studies

Identification of studies relevant to this review will be achieved by searching electronic databases of published literature. For this review, we will search: 1.) PsychINFO, 2.) CINAHL, 3.) Medline, and 4.) Embase. These databases were chosen due to their relevance to the chosen topic. A University librarian was employed to assist with the formation of the search strategy as recommended by McGowan *et al.* (2020). Each strategy was amended to the database-specified requirements in relation to MeSH headings, Boolean operators, and truncation markers. A sample search strategy can be found in Table 2. The study team further agreed to hand-search the reference list of included studies to increase rigour of the search. A grey literature search will be completed to identify potentially missed studies that meet the inclusion criteria via “Google Scholar” and “web searches”. This is common practice in scoping review methods and the authors will search the first hundred results from identified key words and assess eligibility.

**Table 2.** Search strategy.

Search	Search terms
**#1**	(transgender OR gender varian* OR gender conform* OR gender nonconform* OR gender identit* OR gender fluid* OR gender flex* OR gender dysphori* OR gender express* OR gender atypical OR gender bend* OR gender creative* OR gender independen* OR intersex* OR two-spirit OR genderqueer OR nonbinary OR non-binary OR MtF OR FtM OR transexual*)
**#2**	(healthcare OR health* OR health service* OR healthcare delivery OR health service accessibility OR primary health* OR hospital* OR gender care OR gender clinic* OR gender service OR gender reaffirm* OR gender affirm* OR hormone therap* or gender surger* OR transgender healthcare OR trans* health* OR trans healthcare OR transitional care)
**#3**	(quantitative OR survey OR health survey OR questionnaire)
**#4**	S1 AND S2 AND S3

To the best of the authors’ knowledge, no scoping review has been completed on this topic and the findings will seek to add novel data to the current evidence base.

### Stage three: Selection of eligible studies

The review process will consist of two stages. Stage one will be a review of titles and abstracts against an agreed inclusion and exclusion criteria by two researchers (SK and PH). Both reviewers will screen independently using Rayyan software and their results will then be compared and discussed. Rayyan will remove duplicate articles and assist with the screening process. A third reviewer will be available if an agreement cannot be reached (KN). Prior to this, a pilot test will be completed with a sample of studies (n=50) so that the inclusion and exclusion criteria are clear, and decision-making is consistent.

Stage two will involve a full-text review against the same inclusion and exclusion criteria by (SK and KN). DOS will be available as a third reviewer as needed. A PRISMA flow diagram will be created to visually represent the process and ensure transparency. 

The process of study selection will be reported using the Preferred Reporting Items for Systematic reviews and Meta-Analysis extension for Scoping Reviews (PRISMA-ScR) ( Tricco *et al.*, 2018).

### Stage four: Charting the data

SK created a data extraction form that will be used to extract data from the chosen studies as per JBI guidelines (2015) and Peters *et al.* (2020). Data will be charted in Microsoft Excel. Updates and modifications to the data extraction may be added as needed allowing for necessary evolution as data emerges. Modifications may emerge during full text review (by SK and KN) and if a new category is to be added, we will report this in the methods section of review paper and consult with the study team prior (DOS, PH) with rationale.

Initially, the following categories will be charted:

1.) Author and year
2.) Study title, Study aim
3.) Country/region
4.) Population
5.) Sample size
6.) Sample age
7.) Youth included (Y/N)
8.) Study design
9.) Centralised/Decentralised MOC
10.) Theoretical underpinning (if available)
11.) Healthcare access as a primary research topic or subsection
12.) Factors assessed
13.) Instrument development
14.) PPI in instrument development
15.) Availability of full instrument
16.) Overall findings

### Stage five: Collating, summarising and reporting of the results

Extracted data will be collated using Excel and will be shared among the research team for review and final sign-off. Results will be summarised in a narrative manner in keeping with scoping review guidelines. The aim of a scoping review is to provide a broad overview of the findings and therefore a quality assessment will be outside the scope of this project. The results will be reported as a report where thematic analysis will summarise the extracted data in response to the scoping reviews aims, for example, the inclusion of patient and public involvement in instrument design may be discussed as a finding. Importantly, gaps in literature will be reported and direction for future research identified.

### Stage six: Consultation and dissemination

The lead researcher will discuss the findings with an expert panel of transgender and non-binary youth (n=10). They will be consulted regarding dissemination and included where appropriate. In addition, the findings will be discussed with clinicians who work in the provision of gender care. Regarding dissemination, the authors will aim to publish the protocol and findings in peer-reviewed academic journals. In addition, the results will be presented at LGBT conferences (nationally and internationally) and shared with relevant stakeholders and policymakers in the area.

## Conclusion

The results of this scoping review will identify instruments utilised in assessing healthcare access for transgender and non-binary individuals. It will seek to categorise the areas identified as influencing healthcare access. The findings from this study will contribute to the literature and will help to inform future studies looking at barriers to gender-related healthcare in this population.

## Study status

This study is at Stage 2 – a preliminary search of the literature has been conducted and the software packages Mendeley and Rayyan have been trialled.

## Data Availability

No data are associated with this article.
