# Supplementary material for: Instruments used to assess gender-affirming healthcare access: a scoping review protocol
Source: HRB Open Res. 2023 May 19;6:14. Originally published 2023 Feb 20. [Version 3] doi: 10.12688/hrbopenres.13689.3 (PMC10293796; doi:10.12688/hrbopenres.13689.3)
Supplement: Peer review history

# Peer review: Reviewer response for version 3

**DOI:** 10.21956/hrbopenres.15035.r33965

**Published:** 2023-06-26

**Authors:** Beatrice Scholtes, Maxence Ouafik

**Affiliations:** 1 Research Unit of Primary Care and Health, Department of General Medicine, Faculty of Medicine, University of Liège, Liège, Belgium

This new version adequately addresses our concerns.

Is the study design appropriate for the research question?

Partly

Is the rationale for, and objectives of, the study clearly described?

Yes

Are sufficient details of the methods provided to allow replication by others?

Yes

Are the datasets clearly presented in a useable and accessible format?

Not applicable

Reviewer Expertise:

Public health, primary care, organisation of care, general practice, social inequalities, sexual and gender minorities

We confirm that we have read this submission and believe that we have an appropriate level of expertise to confirm that it is of an acceptable scientific standard.

# Peer review: Reviewer response for version 3

**DOI:** 10.21956/hrbopenres.15035.r33964

**Published:** 2023-05-23

**Authors:** András Költő

**Affiliations:** 1 Health Promotion Research Centre, School of Health Sciences, National University of Ireland Galway, Galway, Ireland

The authors adequately addressed all my queries and from my point, the status of the article can be changed to Approved.

Is the study design appropriate for the research question?

Yes

Is the rationale for, and objectives of, the study clearly described?

Yes

Are sufficient details of the methods provided to allow replication by others?

Yes

Are the datasets clearly presented in a useable and accessible format?

Not applicable

Reviewer Expertise:

Health promotion; Health psychology; Adolescent sexual and mental health; Health of gender and sexual minority youth

I confirm that I have read this submission and believe that I have an appropriate level of expertise to confirm that it is of an acceptable scientific standard.

# Peer review: Reviewer response for version 2

**DOI:** 10.21956/hrbopenres.15027.r33560

**Published:** 2023-05-02

**Authors:** András Költő

**Affiliations:** 1 Health Promotion Research Centre, School of Health Sciences, National University of Ireland Galway, Galway, Ireland

The present study is a protocol for a scoping review that aims to collate and synthesise peer-reviewed academic publications and grey literature on the instruments assessing healthcare-related experiences of gender minority individuals who seek gender-affirmative healthcare. The authors propose a six-stage procedure informed by the PRISMA-ScR checklist. These include identifying the research question, identifying and selecting relevant studies, extraction of relevant information, creating a summary of the results and consulting a stakeholder group.

 This is a comprehensive approach which is in line with methodological guidelines for scoping reviews, and the description of the procedure enables fellow researchers to replicate the study. The lessons learnt from this exercise will feed into a new mixed-method study on the experiences of gender minority youth in Ireland, which would remedy a substantial knowledge gap and has the potential to inform decision-making and healthcare services on becoming more inclusive and affirmative of gender minority youth. The method is clearly outlined and properly justified, although the theoretical underpinning would benefit from a more balanced approach that considers gender minority youth’s resilience and other positive factors that enable and empower them to cope with medical and social transition.

 ABSTRACT

 The last sentence of the ‘Background’ paragraph is long, convoluted and needs many reads to understand. Breaking it into two or even three shorter sentences would be helpful.

 There is a contradiction between Background (where a synthesis of peer-reviewed, published academic literature is promised) and the Methods where the search is extended to grey literature sources.

 INTRODUCTION

 The intro is concise and gives a good overview of the theoretical underpinnings of gender minority youth’s experiences with healthcare. A point missing from the overview is what factors are protective to GMY and how their psychological resources (e.g. resilience and hardiness) can contribute to them successfully navigating their journey in gender care.

 The reader would also benefit from a paragraph where you list what are the most common topics/experiences of GMY for which measures exist in the literature, and based on the theoretical points or your observations, what are the areas which lack standardised measures. I appreciate this is the actual aim of your study, but even a subjective account of the existing and lacking measures would help orienting the reader.  

 METHODS

 The authors state: “There is an optional sixth stage which involves consulting with key stakeholders. The research team feels consultation is crucial with populations that are often omitted from research that studies them. The research team will therefore include this sixth stage and utilise an already-established expert panel to consult with key stakeholders.” The two sentences contradict: the second suggests that the stakeholder consultation will happen in any case – then the world “optional” should be deleted from the first sentence.

 Regarding adding grey literature, “This is common practice in scoping review methods and the authors will search the first hundred results from identified key words and assess eligibility.” – I feel searching the first hundred results sounds a bit arbitrary threshold. What if you find 300 records that might be relevant? If combing through the first hundred results is something that is routinely used in previous studies, they should be cited here; otherwise I would recommend being more flexible here, especially that you set no time limit for identifying relevant evidence.

 The inclusion/exclusion criteria and the search strategy are clear and fit for purpose. A comprehensive charting strategy is offered. While I understand that the primary objective is identifying and collating all available measures, I feel adding a methodological/psychometric quality assessment would increase the value of your work. The Revised EFPA Review Model (Evers et al. 2013) ^1^ or Koocher & Rey-Casserly (2013) ^2^ can particularly be helpful in this regard; also check the relevant APA Guidelines ( https://www.apa.org/science/programs/testing/standards). Adding this aspect to the scoping will be largely informative on what is the quality of the available measures and which tools need further validation work.

 CONCLUSION

 A paragraph on how the results of the scoping review will inform your mixed-method study and what do you want to achieve with that (i.e. the mixed-method study) would give broader perspective to the protocol and serve as a reminder to both the reader and the author group what is the worth of carrying out the scoping review. It would also be beneficial to outline how young people and other stakeholders (most importantly parents and healthcare providers) will benefit from the study.

Is the study design appropriate for the research question?

Yes

Is the rationale for, and objectives of, the study clearly described?

Yes

Are sufficient details of the methods provided to allow replication by others?

Yes

Are the datasets clearly presented in a useable and accessible format?

Not applicable

Reviewer Expertise:

Health promotion; Health psychology; Adolescent sexual and mental health; Health of gender and sexual minority youth

I confirm that I have read this submission and believe that I have an appropriate level of expertise to confirm that it is of an acceptable scientific standard, however I have significant reservations, as outlined above.

## References

[1] : Assessing the quality of tests: revision of the EFPA review model. *Psicothema* .2013;25(3) : 10.7334/psicothema2013.97 283-91 10.7334/psicothema2013.97 23910740

[2] : Assessing the Quality of a Psychological Testing Report.2013; 10.1093/med:psych/9780199845491.003.0029 153-154 10.1093/med:psych/9780199845491.003.0029

# Peer review: Reviewer response for version 1

**DOI:** 10.21956/hrbopenres.14970.r33565

**Published:** 2023-04-17

**Authors:** Beatrice Scholtes, Maxence Ouafik

**Affiliations:** 1 Research Unit of Primary Care and Health, Department of General Medicine, Faculty of Medicine, University of Liège, Liège, Belgium

Thank you for the opportunity to review this scoping review protocol. The protocol describes how the authors intend to conduct their scoping review on the topic of access to gender-affirming healthcare. The study aims to identify, explore and map the existing literature concerning healthcare access for transgender and non-binary individuals. The authors have elaborated the following research question:  " *What factors help and hinder access to gender-related healthcare and how are these factors identified by quantitative instruments?".*

 The protocol is well-constructed, the rationale is clear and the objectives well described. The authors have taken into account the different guidelines for scoping reviews. Additionally there is a strong focus on patient and public involvement. Nevertheless we have a few points of concern that we have elaborated on below:

 In the introduction, the authors state that this care is best delivered in a multidisciplinary manner. Could they elaborate on this, particularly on the setting in which they envisage such care? The rationale seems to be that this supportive work is delivered across the lifespan, which is a characteristic of primary care and not necessarily a characteristic of multidisciplinary work per se.

 Furthermore, while there is little evidence on what would constitute the optimal model of care, some qualitative papers on patient preferences do exist. See for example Ker A, Fraser G, Fleming T, *et al.* ‘A little bubble of utopia’: constructions of a primary care-based pilot clinic providing gender affirming hormone therapy. ^1^ and Lee JL, Huffman M, Rattray NA, *et al.* “I Don’t Want to Spend the Rest of my Life Only Going to a Gender Wellness Clinic”: Healthcare Experiences of Patients of a Comprehensive Transgender Clinic ^2^.

 We are concerned that the search strategy may need refining to make it more specific. We tried it in Pubmed and returned more than 65,000 hits. It might be necessary to review and refine it with a librarian. One additional element of concern is that 'intersex*' is part of the search strategy, while constituting a different population than the studied population.

 The inclusion criteria indicates that qualitative data will be excluded. The first part of the research question is relatively qualitative in nature, given that it asks about promoting and hindering factors we wonder if the authors may regret this exclusion since so much nuance is found in qualitative analyses. This may be something that could be assessed during stage 2 of the review. On the other hand, it seems that the authors have already completed a systematic qualitative review so perhaps these results will be pooled. We hope there is enough quantitative data available to feed into this review.

 During stage 3 the authors do not mention how they will evaluate the results from the different reviewers. They could consider the option to calculate a Kappa inter-rater reliability score to check that they are reviewing in a similar way, this would allow them to adjust the process if necessary.

 In stage 4 we would suggest splitting point 2 study title and aim since these are often somewhat different.

 In stage 5 it would be nice if the PPI panel could participate as well. Their appraisal of the information could be valuable at this point.

 A minor point, at the end of paragraph 5 do the authors mean assessed or accessed? *"The study will seek to explore and understand how healthcare is assessed in this population."*

 In general we feel this is an interesting scoping review and will provide helpful insights.

Is the study design appropriate for the research question?

Partly

Is the rationale for, and objectives of, the study clearly described?

Yes

Are sufficient details of the methods provided to allow replication by others?

Yes

Are the datasets clearly presented in a useable and accessible format?

Not applicable

Reviewer Expertise:

Public health, primary care, organisation of care, general practice, social inequalities, sexual and gender minorities

We confirm that we have read this submission and believe that we have an appropriate level of expertise to confirm that it is of an acceptable scientific standard, however we have significant reservations, as outlined above.

## References

[1] : 'A little bubble of utopia': constructions of a primary care-based pilot clinic providing gender affirming hormone therapy. *Health Sociol Rev* .2021;30(1) : 10.1080/14461242.2020.1855999 25-40 10.1080/14461242.2020.1855999 33622204

[2] : *J Gen Intern Med* .2022;37(13) : 10.1007/s11606-022-07408-5 3396-3403 10.1007/s11606-022-07408-5 35112278PMC8809217
